# Attractive and repulsive forces in a crystal of [Rb(18-crown-6)][SbCl_6_] under high pressure

**DOI:** 10.1107/S2052520624001586

**Published:** 2024-03-20

**Authors:** Eduard B. Rusanov, Michael D. Wörle, Maksym V. Kovalenko, Kostiantyn V. Domasevitch, Julia A. Rusanova

**Affiliations:** a Department of Chemistry and Applied Biosciences, ETH Zürich, Vladimir-Prelog-Weg 1-5/10, Zürich, 8093, Switzerland; b Department of Physicochemical Investigation, Institute of Organic Chemistry at National Academy of Sciences of Ukraine, 5 Akademik Kukhar Str., Kyiv, 02660, Ukraine; c Inorganic Chemistry Department, Taras Shevchenko National University of Kyiv, 12, Lva Tolstogo Str., Kyiv, 01601, Ukraine; Politecnico di Milano, Italy

**Keywords:** X-ray diffraction, high pressure, crown ether, attractive and repulsive forces, alkali metal complexes

## Abstract

The compression behavior of [Rb(18-crown-6)][SbCl_6_] crystal under pressure up to 2.16 (3) GPa was investigated in a diamond anvil cell (DAC) using a mixture of pentane–iso­pentane (1:4) as the pressure-transmitting fluid. The compound crystallizes in trigonal space group *R* 3 and no phase transition was observed in the indicated pressure range. The low value of pressure bulk modulus [9.1 (5) GPa] found in this crystal is a characteristic of soft materials with predominant dispersive and electrostatic interaction forces. The nonlinear relationship between unit-cell parameters under high pressure is attributed to the influence of reduced intermolecular H⋯Cl contacts under pressure over 0.73 GPa. It also explains the high compression efficiency of [Rb(18-crown-6)][SbCl_6_] crystals at relatively low pressures, resulting in a significant shift of the Rb atom to the center of the crown ether cavity. At pressures above 0.9 GPa, steric repulsion forces begin to play a remarkable role, since an increasing number of interatomic H⋯Cl and H⋯H contacts become shorter than the sum of their van der Waals (vdW) radii. Below 0.9 GPa, both unit-cell parameter dependences (*P*–*a* and *P*–*c*) exhibit hysteresis upon pressure release, demonstrating their influence on the disordered model of Rb atoms. The void reduction under pressure also demonstrates two linear sections with the inflection point at 0.9 GPa. Compression of the crystal is accompanied by a significant decrease in the volume of the voids, leading to the rapid approach of Rb atoms to the center of the crown ether cavity. For the Rb atom to penetrate into the center of the crown ether cavity in [Rb(18-crown-6)][SbCl_6_], it is necessary to apply a pressure of about 2.5 GPa to disrupt the balance of atomic forces in the crystal. This sample serves as a compression model demonstrating the influence of both attractive and repulsive forces on the change in unit-cell parameters under pressure.

## Abstract 2

The structure of [Rb(18-crown-6)][SbCl_6_] is studied under high pressure. This sample serves as a compression model, demonstrating the influence of both attractive London dispersion forces and repulsive forces in the crystal affecting its behavior under extreme pressure.

## 1. Introduction

**Figure 1:**
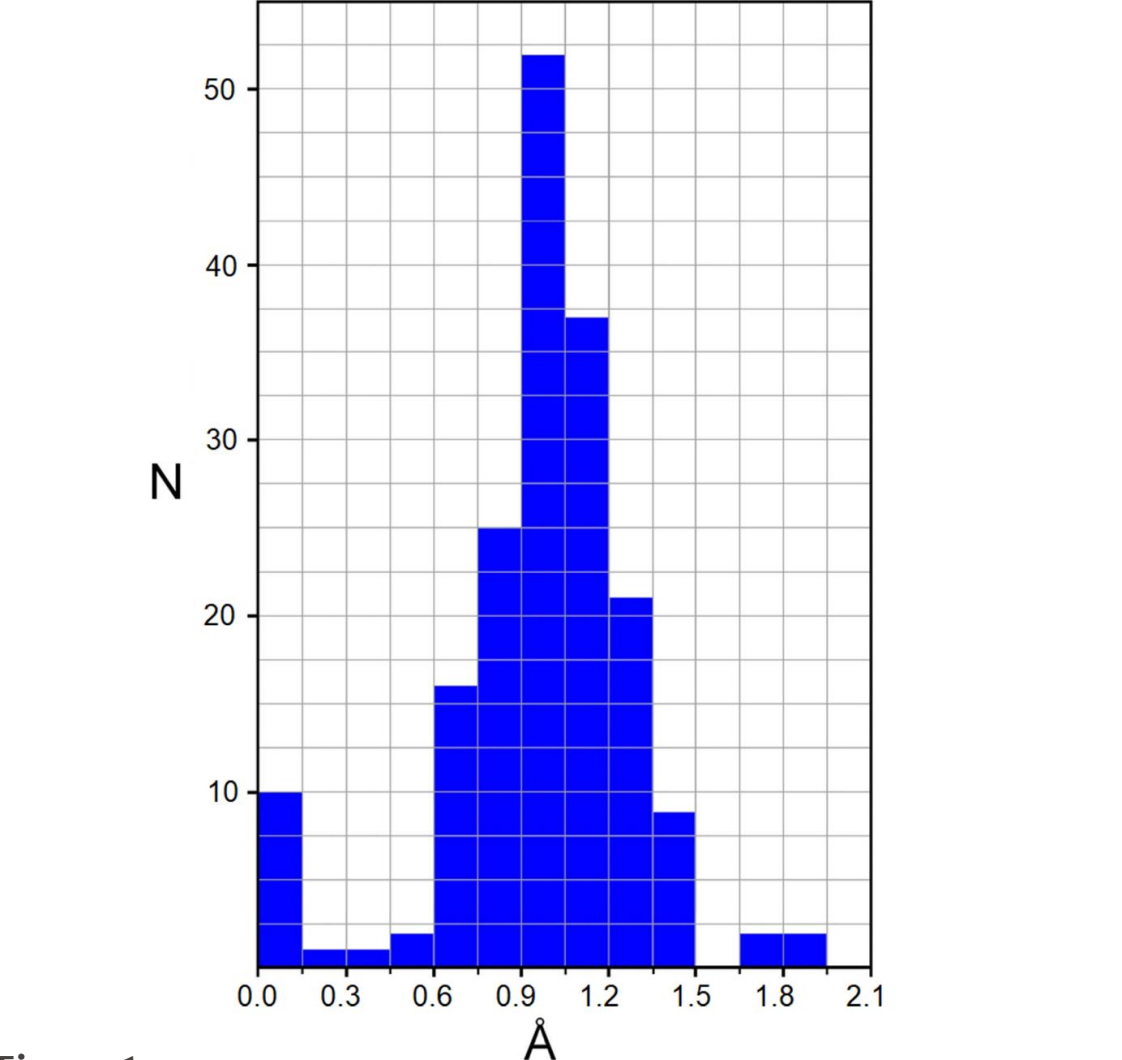
Histogram of frequency of observations the Rb^+^ cation above the mean-square plane of six oxygen atoms of 18-crown-6 according to data from a CCDC search.

Alkali metal ion complexes with highly symmetrical polyether 18-crown-6 are excellent molecular building blocks for solid state design of ionic materials (Steed, 2001 ▸). They are particularly promising in the development of halide perovskites for applications in optoelectronic devices (Zhu *et al.*, 2022 ▸; Xie *et al.*, 2022 ▸), since the coordination geometries adopted by metal halide ionic octahedra and [*M*(18-crown-6)]^+^ counterparts are readily tuneable when varying ionic radii of the encapsulated *M* ^+^ cations (Ferdowsi *et al.*, 2021 ▸; Morad *et al.*, 2020 ▸). In this view, the inherent solid state structural trends of [*M*(18-crown-6)]^+^ complexes are primarily important. Unlike the [K(18-crown-6)]^+^ species, often showing a perfect fit of relatively small K^+^ cations inside the macrocyclic cage (Steed, 2001 ▸), much larger Cs^+^ ions commonly sit far above the ligand plane and, therefore, they are readily accessible for interactions with counter anions. The solid state behavior of the Rb^+^ systems is less comprehensible. At first glance, the rubidium ions closely approximate the behavior of Cs^+^, in most crystal structures usually located 0.9–1.2 Å above the crown ether plane. However, a histogram showing the frequency of observations in the Cambridge Structural Database (CSD) (Groom *et al.*, 2016 ▸) allows us to distinguish also a perceptibly populated group of outliers, which represent 10 examples of complete encapsulation (see Fig. 1 ▸).

Such dual behavior of Rb^+^ ions as either potassium or caesium mimics is rather an intriguing feature that may be suited for the construction of supramolecular switches. The centrosymmetric structure of [Rb(18-crown-6)]^+^ is actualized when: (i) the discoid macrocyclic cations are embedded into chains alternately with centrosymmetric singly charged counter anions [AuCl_4_^−^ (Manskaya *et al.*, 1998 ▸), SbCl_6_^−^ (Ponomarova *et al.*, 2015 ▸) and hydrogen oximate (Domasevitch *et al.*, 1996 ▸)]; (ii) it resides between two large planar moieties (Spisak *et al.*, 2011 ▸; Zabula *et al.*, 2014 ▸; Rusanova *et al.*, 2000 ▸); (iii) in the crystal partial isomorphic substitution in mixed-metal K^+^/Rb^+^ systems is observed (Wallach *et al.*, 2020 ▸). Each of these conditions falls into a category of forced geometries and, therefore, the perfect fit of Rb^+^ ions inside the 18-crown-6 cavity in known structures inevitably relied on ‘pressure of the crystal environment’. However, a similar structural output may be expected also from the forces of the external stimuli, which themselves are simpler to understand and measure. Extreme conditions, such as high pressure in the GPa range, can be considered particularly promising for studies to assess relationships in the system. As a matter of fact, as the Rb^+^ ion virtually approaches the center of the macrocycle, the bond lengths shorten, and the sizes of voids present in the structure decrease. This directly parallels probable primary effects of high-pressure compression. One can anticipate a clear structural response of the [Rb(18-crown-6)]^+^ system to such extreme conditions, with the simple parameter of [Rb(18-crown-6)]^+^ center separation as a function of the applied pressure.

Pressure, traditionally less widely used due to technical/experimental challenges, has become a powerful tool for tuning the properties of different crystals (Woodall *et al.*, 2016 ▸; Poręba *et al.*, 2019 ▸). Unlike temperature, which typically leads to smaller changes, pressure can significantly modify inter­atomic and intermolecular interactions in crystals, and even alter their structure, acting as a key thermodynamic variable. This makes it a highly sought-after tool for research groups exploring new materials. Pressure primarily affects weak non-covalent interactions within the crystal structure, including CH⋯*X* (*X* = metal, halogen) (Moszczyńska & Katrusiak, 2022 ▸; Moszczyńska *et al.*, 2023 ▸; Podsiadło *et al.*, 2014 ▸), halogen⋯halogen (Espallargas *et al.*, 2008 ▸) and H⋯H interactions (Bujak *et al.*, 2016 ▸; Fu *et al.*, 2023 ▸). These modifications are often more pronounced than those achieved through temperature manipulation (Katrusiak, 2019 ▸). However, it is important to note that pressure rarely acts uniformly on crystals. In most cases, it introduces an anisotropic strain, meaning different crystallographic directions experience varying degrees of compression. This can be advantageous for studying non-covalent interactions, as analyzing these variations provides valuable insights (Orgzall *et al.*, 2008 ▸). Such anisotropy can also induce phase transitions at certain pressure points where the system minimizes interactions between closely packed molecules (Giordano *et al.*, 2019 ▸).

In the present work, we explore this new attractive possibility with a closer look at the previously reported [Rb(18-crown-6)][SbCl_6_] complex (Ponomarova *et al.*, 2015 ▸), which was selected for a range of particular features. Within the one-dimensional cation–anion chains, the [Rb(18-crown-6)]^+^ moieties are centrosymmetric, with the Rb^+^ ion equally disordered over two positions around the center of 18-crown-6 at a relatively small distance of about 0.5 Å. Thus, the separation between the two positions of the disordered Rb^+^ ions, which is twice the distance between the crown ether center and the metal, is a good indicator for further evaluation of pressure effects. The high metric symmetry of the crystal (trigonal 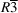 ) is also valuable, as it facilitates the accumulation of higher completeness for diffraction experiments in a high-pressure (HP) diamond anvil cell. In addition, the completely ordered structure of the crown ether in the crystal is beneficial for the diffraction capability of the compound at room temperature.

## 2. Experimental

### 2.1. Preparing the crystal and the DAC for the experiment

The [Rb(18-crown-6)][SbCl_6_] complex was synthesized and crystallized from di­methyl­formamide following the literature method (Ponomarova *et al.*, 2015 ▸). Before the high-pressure (HP) experiments, several crystals of the material were tested on the diffractometer for the best quality diffraction criteria with sharp spots at the highest diffraction angles. For the selected good quality crystal with dimensions of 0.11 mm × 0.12 mm × 0.15 mm, the preliminary diffraction experiments performed at low (100 K) as well as at ambient temperatures [293 (1) K] proved satisfactory diffraction for subsequent experiments in a diamond anvil cell. The compression of a single crystal in the experiment was carried out using a Diacell Tozer diamond anvil cell (DAC) supplied by Almax EasyLab with an opening angle of 41° equipped with Boehler–Almax cut diamonds with 0.8 mm culets. For the experiments, the gaskets, made of 0.25 mm BeCu alloy foil, were pre-indented to a thickness of 0.17–0.20 mm. A spark-eroded hole, created using Loto Eng. Spark E400, with a diameter of 0.30–0.35 mm, was used.

In a series of HP diffraction experiments [at pressures of 0.15 (3), 0.29 (3), 0.38 (3), 0.60 (3), 0.68 (3), 0.83 (3), 0.92 (3), 0.98 (3), 1.13 (3), 12.5 (3), 14.6 (3), 16.1 (3) and 1.89 (3) GPa], a mixture of pentane–iso­pentane (1:4) was used as a pressure-transmitting medium. This hydro­static medium is very volatile (bp ∼35°C), therefore, the DAC and hydro­static medium were cooled to −17°C in a fridge before loading. The crystal and ruby ball were glued to the DAC culet using a minimum amount of transparent ep­oxy resin without hardener. The cell was loaded with the selected crystal, reference ruby ball for the pressure control and pressure-transmitting medium, then the DAC was immediately sealed. After visual inspection for the absence of air bubbles inside, the DAC was placed into a T-press (LOTO-eng.) for compression and the step-by-step load to the Tozer DAC was applied. The pressure inside the DAC was monitored before and after each diffraction experiment by the ruby fluorescence pressure measurement method (Piermarini *et al.*, 1975 ▸) with an accuracy of ±0.03 GPa.

To obtain high-quality diffraction data from large crystals with high *I*/σ(*I*), we employed a metal gasket of considerable thickness and a large-diameter hole. This resulted in an expansion of the working chamber volume and an outward displacement of the gasket at pressures of around 1.8–2 GPa. Fearing a possible breakthrough of the HP liquid and crushing of the crystal under further compression, we did not perform experiments at higher pressures. In addition, measurements were taken during pressure release at four different pressure points [1.23 (3), 0.95 (3), 0.76 (3) and 0.30 (3) GPa]. After pressure release the crystal was extracted from the DAC and used again for unit-cell parameters and structure determination at ambient conditions. The pressure control with the following data collection on the diffractometer was performed with a delay for 15 min after pressure rise and for 1 h after pressure release. Data collection for the crystal extracted from the DAC after HP experiments was performed the next day.

To perform the HP experiment, the nozzle of the cryo-system was lifted and a single-cup beamstop with a larger crystal-to-beamstop distance was installed to create enough space for the HP cell. The crystal-to-detector distance was set up to 60 mm. Then a Victrex PEEK 450g holder together with the high-pressure Tozer DAC was fixed into the goniometer head and the crystal was readily centered on the diffractometer (Dera & Katrusiak, 1999 ▸; King & Finger, 1979 ▸).

### 2.2. Data collection, reduction and refinement

The diffraction data were collected at 293 K on a Rigaku XtaLAB Synergy-S diffractometer equipped with a Photon­Jet-S microfocus Mo X-ray source with a mirror design and a hybrid photon-counting HyPix-6000HE detector. An exposure time of 4–7 seconds per degree was estimated as the target for the data collection. The processing procedures for the HP experiments to a resolution of 0.75 Å were followed by procedures implemented in *CrysAlis Pro* software (Agilent, 2014 ▸). The integrations were carried out using dynamic masking of the regions of the detector shaded by the HP cell. During the data reduction, different maximum opening angles for the HP cell were applied. The opening angle of 41° used in *CrysAlis Pro* for data reduction of HP experiments provided slightly better completeness but essentially poorer *R*_int_ and structure refinement parameters. This situation is likely conditioned by some uncertainty in the determination of the intensity of reflections and background around maximal opening angles. Therefore the final data reduction was performed to opening angles of 38°.

The structures were solved by direct methods and refined by the full-matrix least-squares on *F* ^2^ for all reflections using *SHELXL* (Sheldrick, 2015 ▸) operated under *OLEX2* (Dolomanov *et al.*, 2009 ▸). The non-hydrogen atoms were refined freely with anisotropic displacement parameters and with an occupancy factor of 1/6 for Rb1 atom, which sits on threefold axes and is equally disordered about the center of inversion. All H atoms were placed at calculated positions and refined as riding, with C—H = 0.97 Å, and with *U*_iso_(H) = 1.2*U*_eq_(C). Information about the crystal data and main structure refinement details at various pressures are summarized in Table 1 ▸.

## 3. Results and discussion

### 3.1. Ambient-pressure structure

**Figure 2:**
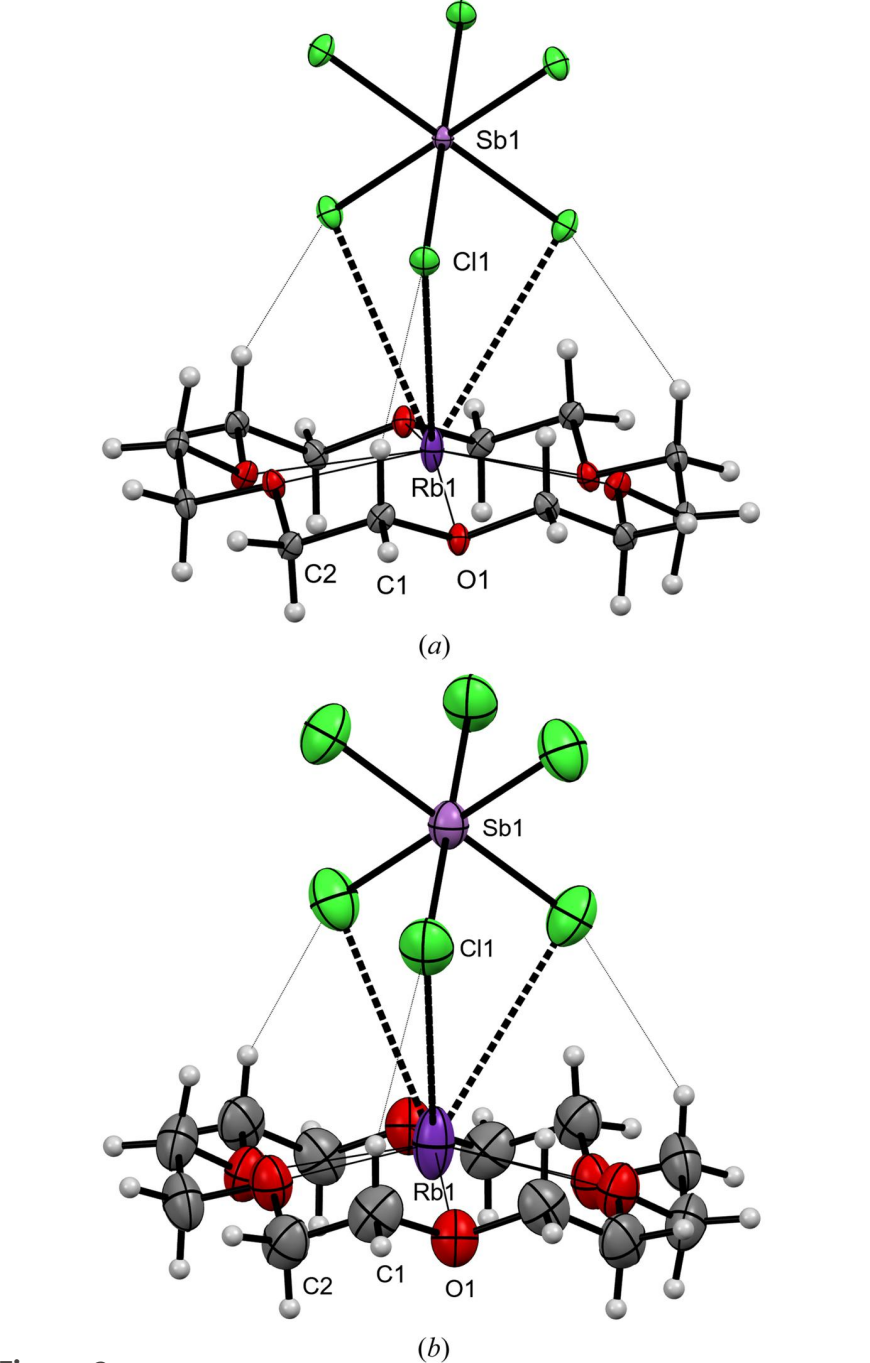
Structure of [Rb(18-crown-6)][SbCl_6_] at 100 K (*a*) and at 293 K (*b*), with the displacement ellipsoids drawn at the 50% probability level. Only independent atoms are labelled.

The title crystal structure comprises centrosymmetric hexachloridoantimonate(V) anions and [Rb(18-crown-6)]^+^ cations (see Fig. 2 ▸). The former are located on a threefold axis, with the Sb atoms situated at centers of inversion and therefore the independent part of the unit cell consists of only one Cl atom in a general position and one-sixth of Sb atoms at special positions. Since the [Rb(18-crown-6)]^+^ cations also reside on a threefold axis passing through the 18-crown-6 centroid, which coincides with a center of inversion, the independent part comprises only of a one-sixth complex moiety. At ambient-pressure conditions, the Rb^+^ cations are equally disordered over a center of inversion and reside at two positions, above and below the plane of the 18-crown-6 ligand defined by six O atoms.

**Figure 3:**
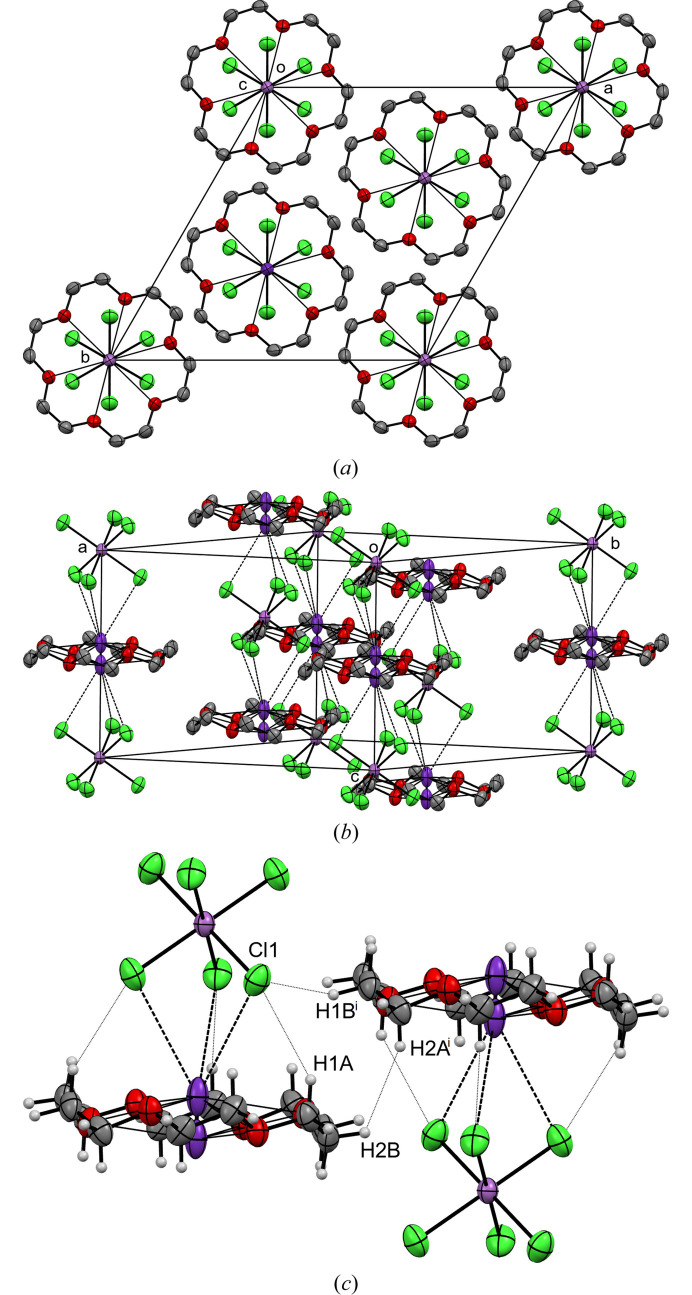
Molecular packing of [Rb(18-crown-6)][SbCl_6_] viewed along the *c* direction (*a*) and the orthographic view of the unit cell (*b*). Hydrogen atoms are omitted for clarity. (*c*) The shortest intra- and intermolecular H⋯Cl and H⋯H contacts. Symmetry code (i): 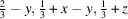 .

According to the symmetry, the crown ether molecule adopts the *D*_3d_ conformation. The packing pattern for this structure is represented by extended chains along the *c*-axis [see Figs. 3 ▸(*a*) and 3 ▸(*b*)], which are sustained by alternating [Rb(18-crown-6)]^+^ cations and SbCl_6_^−^ anions with a set of ion-dipole Rb⋯Cl at 3.7255 (13) Å and very weak C1—H1*a*⋯Cl1 interactions with the following parameters at ambient conditions: H1*a*⋯Cl1 = 3.08 Å, C1—H1*a*⋯Cl1 = 146°. In this work, we will discuss the shortest interactions between CH⋯Cl and H⋯H in a crystal, shown in Fig. 3 ▸(*c*). Additionally, the crystal exhibits weak C1^i^—H1*b* ^i^⋯Cl1 (i = 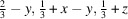 ) interactions, directed in the *ab* plane between adjacent columns, with a H1*b* ^i^⋯Cl1 distance of 3.11 Å and a C1^i^—H1*b* ^i^⋯Cl1 angle of 130°. The intermolecular interactions are further characterized by the presence of the shortest H2*b*⋯H2*a* ^i^ contact between adjacent columns at 2.62 Å. Although all these interactions are longer than the sum of the vdW radii for H/Cl and H/H pairs of atoms (2.95 and 2.40 Å, respectively) (Bondi, 1964 ▸), they nevertheless play an important role in the further discussion of contacts in a crystal under high pressure.

When comparing the results gained at 293 K and at 100 K, and also a previously performed determination at 173 K (Ponomarova *et al.*, 2015 ▸), not only thermal motion and unit-cell parameters but also some geometry parameters of the structure reveal a degree of temperature dependence (see Table 1 ▸). By cooling to 100 K, the Rb1–Cl1 distance as well as the shortest H⋯Cl and H⋯H contacts undergo a perceptible shortening: 3.7255 (13) to 3.6834 (8) Å; 3.08/3.11 to 2.97/3.04 and 2.62 to 2.49 Å, respectively). Considering the extension of the H⋯Cl and H⋯H contacts within the crystal structure where intermolecular distances exceed the sum of their vdW radii, we can conclude that the crystal exhibits a loose packing type (Kaźmierczak & Katrusiak, 2013 ▸). This suggests a structure with significant voids between the molecules of the compound in the crystal.

Since the ionic radii for the Cl^−^ anion is 1.81 Å, and for the Rb^+^ cation with coordination numbers 6, 9 and 12 the typical values are 1.52, 1.63, and 1.72 Å, respectively (Shannon, 1976 ▸), one would expect that the Rb–Cl bond distances should be in the range 3.33–3.53 Å. However, the unusually large Rb–Cl distance of 3.7255 (13) Å observed in the crystal structure of [Rb(18-crown-6)][SbCl_6_] under ambient conditions is notable for its significant elongation of ∼0.2–0.4 Å compared with the corresponding Rb–Cl distances in various compounds according to CSD (Groom *et al.*, 2016 ▸). This Rb–Cl distance is one of the longest ever observed and is comparable to the C–Cl⋯Rb interactions of 3.697 and 3.721 Å found in the crystal of *catena*-[(μ_3_-di­chloro­acetic acid)-(μ_4_-di­chloro­acetato)-rubidium] (Yoshida & Kashino, 1997 ▸) or the weak interactions between Rb^+^ cations and solvate chloro­form molecules (Mäkelä *et al.*, 2016 ▸). This significant increase in Rb–Cl distance suggests that the interaction between rubidium cations and SbCl_6_^−^ anions in the crystal is considerably weakened. An even more important feature concerns cooling to 100 K and the accompanying shortening of the distance between two components of the disordered Rb^+^ cations [1.121 (2) to 0.9078 (14) Å], in contrast to the value 0.962 (2) Å obtained earlier at 173 K (Ponomarova *et al.*, 2015 ▸)]. Thus the Rb^+^ cations tend to approach the center of 18-crown-6 as the temperature decreases. This results in the compression of the structure along the direction of cation–anion chains by 0.294 Å, which is best reflected by 2.9% reduction of the crystallographic *c*-axis length, whereas *a*/*b* axes shrunk only by 0.089 Å or 0.6%.

### 3.2. Response of the unit-cell parameters to applied pressure

**Figure 4:**
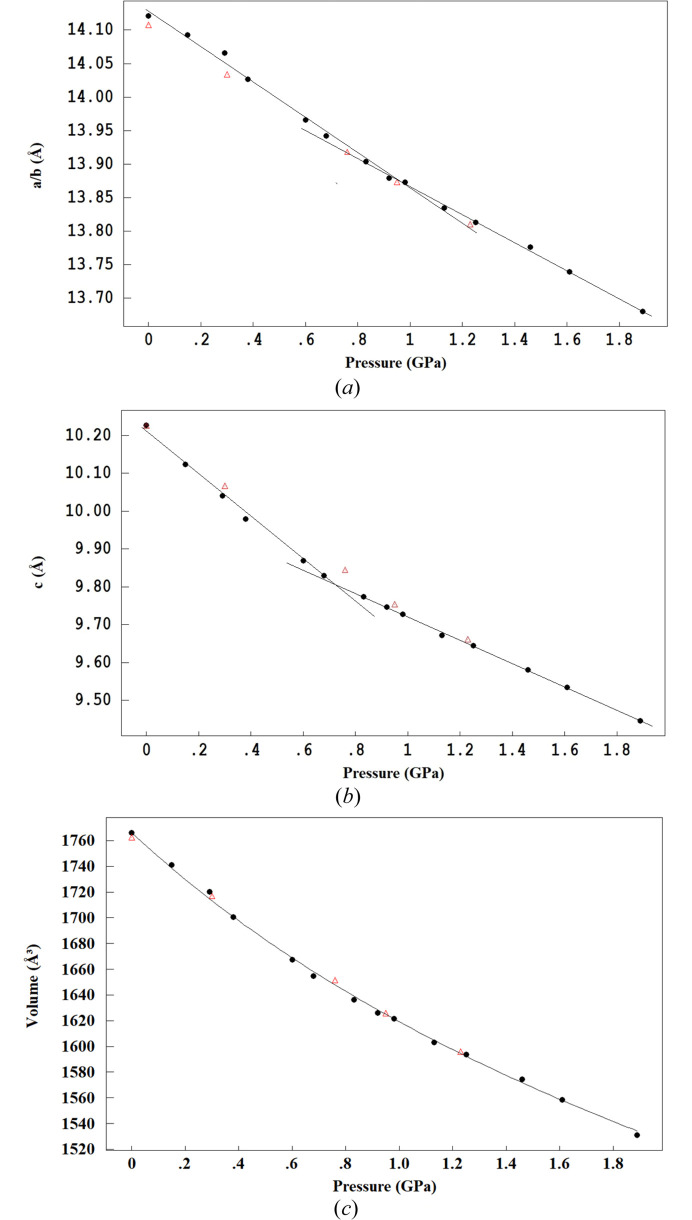
Unit-cell parameters *a* (*a*), *c* (*b*) and *V* (*c*) as a function of pressure in the [Rb(18-crown-6)][SbCl_6_] crystal.

The response of the crystal structure to hydro­static pressure is anisotropic across the pressure range. Among the unit-cell parameters, the *c*-axis is the most compressible, shortening by 7.67% upon increasing pressure up to 1.89 (3) GPa. The *a*- and *b*-axis lengths decreased by 3.12%. The nonlinear relative changes in the unit-cell parameters and unit-cell volume are shown in Figs. 4 ▸(*a*)–4 ▸(*c*). On the graphs are shown data for the crystal with increasing (black circles) and decreasing pressure (red triangles). In Tables 1 ▸ and 2 ▸ data for points with decreasing pressure are marked by the letter r.

The unit-cell volume changes monotonically between the lowest and highest measured pressures with both increasing and decreasing pressures, demonstrating a reversible recovery after decompression, leading to a unique and consistent data set that can be fitted with the same equation of state (EoS).

Therefore, all collected experimental data were combined into a single dataset. The pressure–volume (*P*–*V*) data [Fig. 4 ▸(*c*)] were analyzed using the *EosFit7* software (Gonzalez-Platas *et al.*, 2016 ▸) and the best fit was obtained using a third-order Birch–Murnaghan EoS (Angel, 2000 ▸). The calculated values for zero pressure volume *V*_0_ and pressure bulk modulus *K*_0_ were 1767 (3) Å^3^ and 9.1 (5) GPa, respectively. Pressure derivative *K*′ was 5.3 (8). This relatively small bulk modulus is characteristic of soft molecular materials (Angel, 2004 ▸) such as organic or metal–organic compounds, where intermolecular interactions are mainly dominated by dispersion forces and/or electrostatic interactions.

While the unit-cell parameters *a* and *c* generally exhibit a dependence on pressure as expected from the *P*–*V* graph, a closer examination of the *P*–*c* graph reveals two distinct linear regions durnig compression. These regions occur within the pressure ranges of 0–0.73 and 0.73–1.9 GPa. Despite the lower compressibility of the crystal in the direction of the *ab* plane, the dependence *P*–*a* is also almost linear in two pressure regions of 0–0.95 and 0.95–1.9 GPa, and an inflection point is expected within the pressure range 0.85–1.1 GPa. It is worth noting that the *P*–*V* plot also revealed two perfectly linear sections in the pressure ranges 0–0.7 and 0.7–1.89 GPa.

While the *P*–*V* dependence during compression/decompression cycles matches closely, some hysteresis effect in the pressure dependence is evident for the *P*–*a* and *P*–*c* dependences at pressures below 0.95 GPa. In the cycle of pressure release, the crystallographic *a* axis parameter differs at almost the same pressure values. Thus, at a pressure of 0.30 (3) GPa during the pressure release cycle, the crystallographic *a* axis parameter is approximately 0.032 Å larger than at an almost similar pressure of 0.29 (3) GPa during the pressure compression cycle. This indicates that the unit cell exhibits some memory of its compressed state after decompression. Conversely, a slight increase in the crystallographic *c* axis parameters is observed at points with pressure 0.76 (3) and 0.30 (3) GPa, by approximately 0.043 [extrapolated using data for nearest values at 0.68 (3) and 0.83 (3) GPa for the compression cycle] and 0.027 Å, respectively. It is worth noting that the observed difference in unit-cell parameters in the compression and decompression cycles exceeds the precision of its determination within 3σ.

The observed behavior of the crystal under the decompression cycle indicates that there is more space along the cation–anion chains (voids will be discussed separately) under pressures below 0.9 GPa, and the absence of reduced intermolecular interactions in the crystal allow some variation in unit-cell parameters, although the unit-cell volume remains essentially unchanged. However, above 0.9 GPa, the repulsive forces in the crystal become too strong to allow for significant unit-cell parameter variation, causing the lattice to return to its original dimensions.

### 3.3. The effect of pressure on Cl⋯H and H⋯H contacts in the crystal

In the previous section, from the *P*–*a* and *P*–*c* graphs, we have noted two critical inflection points at 0.73 and 0.95 GPa, which divide the graphs into two separate linear sections with different slopes and it was expected that this may correspond to the appearance of shortened intermolecular contacts in the crystal.

At close intermolecular distances, when all interatomic distances are only slightly greater than the sum of the vdW radii, the attractive London dispersion forces (Eisenschitz & London, 1930 ▸; London, 1930 ▸) and electrostatic interaction forces play an important role in organization of molecular crystals (Corpinot & Bučar, 2019 ▸; Liptrot & Power, 2017 ▸; Das & Datta, 2023 ▸; Hermann *et al.*, 2017 ▸). Further shortening of the contacts under the influence of pressure when the contacts reach the equilibrium point (corresponds to vdW contacts for atomic pairs) dramatically changes the nature of interactions in the crystal from attractive to more strong repulsive forces when electronic shells of atoms are deformed. Thus, presumably, a certain range of pressures corresponding to the appearance of contacts equal to the sum of the vdW radii, an inflection point is revealed by the change in the slope of the corresponding *P*–*a* and *P*–*c* graphs.

It is reasonable to assume that after the critical inflection point, further compression of the structure would be less efficient due to the increasing repulsion between H/Cl and H/H atomic pairs. Further insights into the nature of the structure’s response to pressure were gained through an analysis of non-covalent interactions.

The H1*a*⋯Cl1 interactions between Cl atoms of the SbCl_6_^−^ anion and H atoms of the crown ether in the columns directed along the crystallographic *c*-axis (data from Table 2 ▸) remained relatively long up to a pressure of 0.68 (3) GPa. This pressure corresponds to H⋯Cl contacts of 2.95 Å, which is equivalent to the sum of vdW radii for H/Cl pairs. However, the H⋯Cl contacts between neighboring columns remain greater than the sum of their vdW radii up to pressures of 1.13 (3) GPa. Only at this pressure were additional vdW interactions observed, including additional contact C2—H2*b*⋯H2*a* ^i^ (2.41 Å). At pressures exceeding 1 GPa, stronger interactions between SbCl_6_^−^ anions and the crown ether facilitate the formation of exceptionally short CH⋯Cl and CH⋯HC contacts down to 2.82 and 2.33 Å, respectively. A comprehensive list of short contacts at a pressure of 1.89 (3) GPa in the [Rb(18-crown-6)][SbCl_6_] crystal is presented in Table 3 ▸.

**Figure 5:**
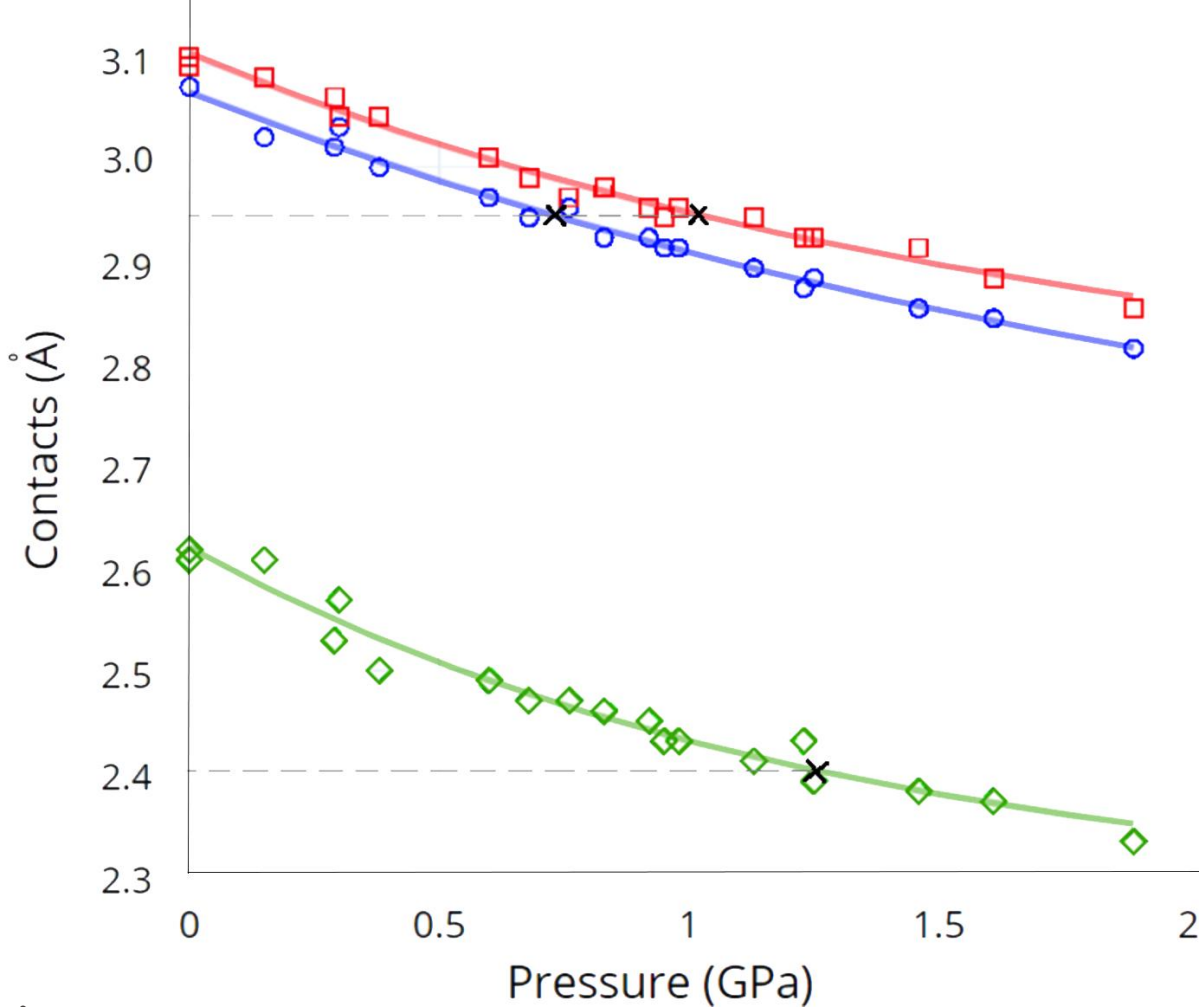
Lengths of H⋯Cl and H⋯H contacts as a function of pressure in [Rb(18-crown-6)][SbCl_6_]. H⋯Cl contacts in columns and between adjacent columns in the *ab* plane are indicated with open blue circles and open red squares, respectively, and H⋯H contacts are marked as open green diamonds. The black crosses indicate the pressure corresponding to the vdW contacts for the H/Cl and H/H atom pairs. The plots were smoothed using an exponential function.

To investigate the dependence of short contacts H⋯Cl and H⋯H on pressure, we plotted the data from Table 2 ▸ on the graph shown in Fig. 5 ▸. The points representing H⋯Cl contacts in columns along the *c*-axis and between neighboring columns in the *ab* plane are indicated in blue circles and red squares, respectively, and the points representing H⋯H contacts are at the bottom and marked in green. All plots were smoothed using an exponential function. Dashed lines parallel to the pressure axis correspond to the vdW interaction distances of 2.95 Å and 2.40 Å for H⋯Cl and H⋯H contacts, respectively. The distance between H and Cl atoms along the *c*-axis, which corresponds to the sum of their vdW radii (2.95 Å), is observed at approximately 0.7–0.75 GPa, as seen in the graph in Fig. 5 ▸. Similarly, analyzing short H⋯Cl contacts between adjacent columns in the *ab* direction reveals that the pressure at which vdW contacts of 2.95 Å are formed corresponds to a pressure slightly above 1.0 GPa. In contrast, the pressure at which H⋯H contacts between adjacent crown ether species approach the sum of their vdW radii (2.40 Å) is significantly higher, at around 1.25 GPa. This suggests that the behavior of H⋯H contacts does not have a major influence on the *P*–*a* relationship.

These findings provide a rationale for the observed trends in the pressure-dependent unit-cell parameters discussed in the previous section, with the inflection point at 0.73 and around 1.0 GPa clearly evident in the corresponding plots. Thus, the presence of inflection points on the *P*–*a* and *P*–*c* plots may be associated with the presence or absence of shortened contacts in the crystal at a certain pressure.

### 3.4. The effect of pressure on the deviation of the Rb^+^ cation from the crown ether cavity.

**Figure 6:**
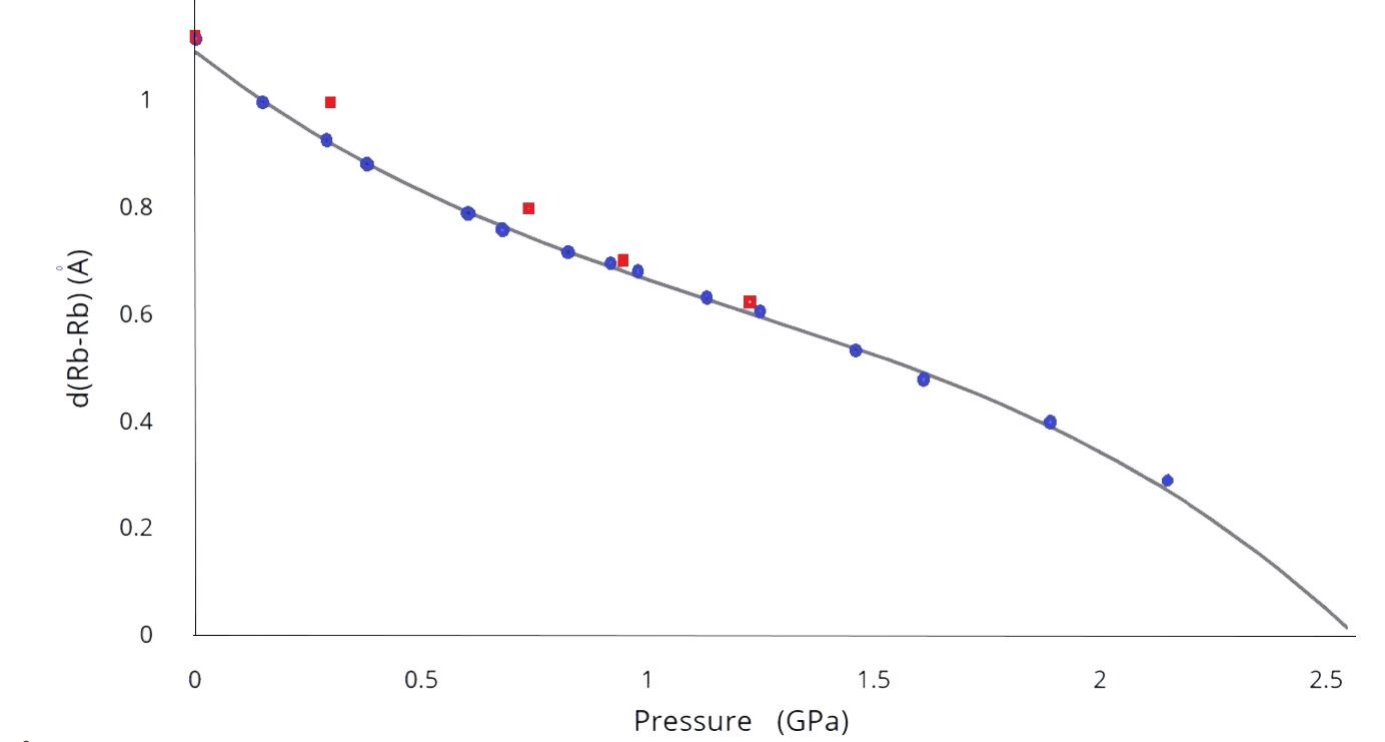
The dependence of the separation between two disordered positions of the Rb atom on pressure in [Rb(18-crown-6)][SbCl_6_]. The best fit approximation is made using the third-order function *P* = *A* + *B*d + *C*d^2^ + *D*d^3^ in pressure range 0–2.16 GPa with *A*, *B*, *C* and *D* coefficients of 1.095, −0.6621, 0.3268 and −0.0915, respectively. *P* = pressure and d = *d*(Rb–Rb) separation. Blue circles and red squares correspond to data in compression and decompression cycles, respectively.

With increasing pressure, the Rb^+^ cations move progressively closer to the crown centers leading to the gradual merging of two symmetry-related disordering components in effect (see data at Table 2 ▸). Within a pressure range of 0–0.29 (3) GPa, the corresponding Rb–Rb separation [*d*(Rb–Rb)] decreased by 0.193 Å from 1.121 (2) Å at ambient conditions to the value of 0.928 (2) Å, then doubling the pressure to 0.6 (3) GPa leads to a decrease of 0.135 Å more to the value of 0.7928 (19) Å. Further compression of the crystal from 0.6 (3) to 1.25 (3) GPa leads the decreased Rb–Rb separation by 0.185 Å only to 0.608 (5) GPa. From these data, it is seen that the pressure–*d*(Rb–Rb) [P–*d*(Rb–Rb)] dependence has a nonlinear form. This trend continued in a pressure range of 1.4–1.9 GPa and it presumably leads to the completely ordered model. At first glance, the pressure dependence of the Rb–Rb separation [*P*–*d*(Rb–Rb)] in the disordering scheme (see Fig. 6 ▸) largely follows the character of the graph for the unit-cell parameters under pressure. However, the fit by the linear or two linear functions in different pressure ranges as well as the exponential function provides some coincidence of the experimental points at many regions of the graph. Nevertheless, the best fit of the dependence was achieved using a third-order function *P* = *A* + *B*d + *C*d^2^ + *D*d^3^ in full pressure range with the following *A*, *B*, *C* and *D* coefficients: 1.0950, −0.6621, 0.3268 and −0.0915. The pressure at which the rubidium atom is placed exactly in the center of the crown ether cavity is achieved at a pressure of ∼2.5 GPa.

For another crystal we performed a series of HP diffraction experiments up to the pressure of 2.16 (3) GPa, but with no pressure release. In general, the set of obtained data duplicates our experimental points, which also includes points at pressure release. Therefore, we decided not to include these data. However, they include an essential point at 2.16 (3) GPa, which corresponds to *d*(Rb–Rb) of only 0.28 Å and also fits well the third-order function for *P*–*d*(Rb–Rb) dependence. Therefore, this point was also included in the plot in Fig. 6 ▸. It is worth noting that as soon as the pressure reaches 1.89 (3) GPa, refinement of the structure becomes almost equally successful for the ordered model with Rb sitting on the inversion center or for the disordered model with *d*(Rb⋯Rb) separation of 0.4 Å. However, this ordered model yields slightly longer Rb—Cl bond distances [3.8541 (11) Å] and exceedingly large ADP values along the *z*-axis for the Rb atoms. A similar situation was observed for the crystal at the pressure of 2.16 (3) GPa, but only the restrained refinement of the disordered model was possible since Rb^+^ cations fall to the center. It can be assumed, however, that at least some of the Rb^+^ cations in the crystal at a pressure around 2 GPa reside in the inversion centers. Generally, the *P*–*d*(Rb–Rb) relationship resembles a linear trend in the pressure range of approximately 0.7–1.9 GPa, so the downward section when approximated by a third-order function above 1.8 GPa requires further explanation.

As the crown ether molecule adopts a non-planar conformation, three oxygen atoms of the crown ether reside slightly above and three slightly below the mean-square plane of these six O atoms, resulting in an interplanar distance in 18-crown-6 in the range of 0.45–0.50 Å under various pressures. Data, presented in Table 2 ▸, clearly demonstrate that under pressure around 1.6–1.9 GPa, the shortest Rb–O distances of 2.827 (2) and 2.822 (3) Å correspond to a disordered model with Rb atoms deviating from the center by 0.2–0.24 Å [*d*(Rb–Rb) = 0.4–0.48 Å]. In contrast, the structure with a centrosymmetric arrangement of rubidium atoms [data at 1.89 (3) GPa presented in Tables 1 ▸ and 2 ▸ and marked with letter c] is characterized by six equivalent but slightly longer Rb–O distances at 2.833 (3) Å. For a rubidium atom with a large ionic radius, this suggests that the steric barrier to entering the cavity of the crown ether is located at a distance of approximately 0.22–0.25 Å from the center. When a rubidium atom, driven by external pressure, enters the cavity of the crown ether between the three closest oxygen atoms, it occurs in the center of the cavity surrounded by six oxygen atoms at equivalent distances. Partial penetration of rubidium atoms through the barrier of the three closest oxygen atoms into the cavity introduces additional statistical disordering of Rb atoms, with some of them located in the center and others outside. This explains the observed strong elongation of the ellipsoid of thermal vibrations for rubidium atoms in the HP range (>1.4 GPa) and the rapid descent observed in the graph in Fig. 6 ▸ at pressures exceeding 2 GPa.

A very remarkable fact is that when the crystal decompressed, hysteresis in the deviation of rubidium atoms from the center of the crown ether was also discovered. Following compression and decompression cycles observed for unit-cell parameters, the values for *d*(Rb–Rb) separation in the HP range are identical within 3σ. At 1.25 (3) and 1.23 (3) GPa in compression and decompression cycles, *d*(Rb–Rb) values are 0.608 (5) and 0.634 (7) Å, respectively. At a lower pressure of 0.95 (3) GPa in the decompression cycle, the Rb–Rb separation increases to 0.705 (9) A, while during crystal compression for the nearest points during crystal compression at 0.92 (3) and 0.98 (3) GPa (mean pressure is 0.95 GPa), the mean value is 0.688 (3) Å. However, after a further decrease in pressure, a significant hysteresis in the deviation of rubidium atoms from the center of the (18-crown-6) is observed. Thus, at 0.76 (3) GPa pressure in the decompression cycle, *d*(Rb–Rb) is 0.804 (4) Å, and this value is 0.065 Å longer than the mean *d*(Rb–Rb) deviation of 0.739 Å found for the nearest points at 0.68 (3) and 0.83 (3) GPa with a mean pressure of 0.755 GPa in the compression cycle. This value even exceeds *d*(Rb–Rb) of 0.7928 (19) Å found at pressure 0.60 (3) GPa in the compression cycle. This trend with hysteresis in the pressure release is observed when the pressure is reduced again to 0.30 (3) GPa, *d*(Rb–Rb) in pressure points 0.30 (3) and 0.29 (3) GPa are 0.992 (4) and 0.928 (2) Å respectively, so the difference of 0.064 Å is significant.

Since the CSD contains data about several structures in which the rubidium atom occupies a position exactly in the center of the 18-crown-6 cavity, one would expect that during the decompression cycle, at least some of the rubidium atoms would remain inside the 18-crown-6 cavity after releasing pressure. However, this does not happen, and when the pressure is released to atmospheric pressure, all structural parameters return to their original state. So this effect with *d*(Rb–Rb) hysteresis can be associated with the hysteresis of the crystallographic *c* axis parameter since it also elongates noticeably in the direction of molecular chains at pressures of 0.76 (3) and 0.30 (3) GPa.

### 3.5. The effect of pressure on interatomic distances in the crystal

Within the pressure range up to 1.89 (3) GPa, the shorter of the two symmetry-independent Rb—O bond distances exhibits a relatively modest decrease by 0.027 Å from 2.8486 (17) to 2.822 (3) Å, while the longer Rb—O bond distances decrease notably by 0.078 Å from 2.9354 (18) to 2.857 (4) Å, leading to an average decrease of 0.053 Å in Rb—O bond distances. The geometry and conformational features of the crown ether remain largely unaffected by pressure, as demonstrated by the unaltered C—O and C—C bond distances and bond angles. Thus, it was found that the C—O and C—C bond distances in the structures at different pressures were in the range of 1.414–1.444 (7) and 1.476–1.501 (9) Å, respectively. Additionally, the largest difference in C—C—O—C and O—C—C—O torsion angle values between the structure at normal pressure and 1.89 (3) GPa do not exceed 3°.

The geometry of the SbCl_6_^−^ anion remains largely unaffected by pressure, with Sb—Cl bond distances remaining within a narrow range of 2.3641 (10)–2.3769 (13) Å, resulting in a maximum shortening of bond lengths by 0.013 Å only. Analysis of Rb—Cl bond lengths shows that even at high pressure these bonds are in a fairly narrow range of values of 3.684 (2)–3.7304 (17) Å. Similar Sb—Cl and Rb—Cl bond lengths (for Rb atom with coordination number of 12) in the range 2.368–2.374 Å and 3.536–3.656 Å, respectively, have been reported for the inorganic salt Rb^+^SbCl_6_^−^ (Benin *et al.*, 2021 ▸). However, these Rb—Cl bond lengths are significantly longer than those found in related complexes such as [Rb(18-crown-6)][SbCl_5_][Rb(18-crown-6)] and [Rb(18-crown-6)][*E*Cl_6_][Rb(18-crown-6)] (where *E* = Sn, Te) (3.328–3.433 Å) (Morad *et al.*, 2020 ▸; Zhu *et al.*, 2022 ▸), or in hybrid perovskite materials [cat]^2+^[RbCl_3_]^2−^ with different organic cations (3.197–3.318 Å) (Paton & Harrison, 2010 ▸; Zhang *et al.*, 2017 ▸). These long distances indicate a relatively weak interaction between rubidium cations and the chlorine atoms in the crystal.

### 3.6. Voids analysis

**Figure 7:**
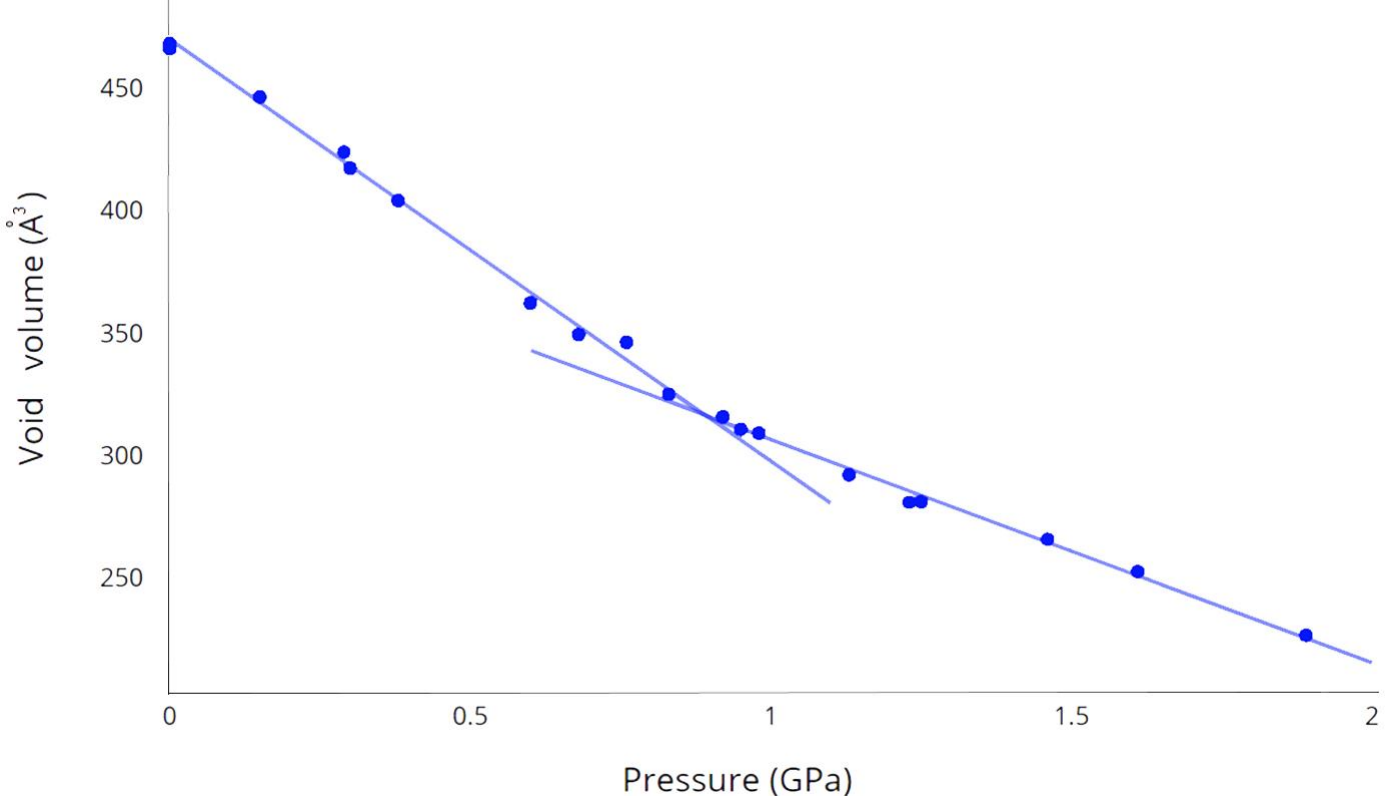
Total void volume at different pressures in a crystal of [Rb(18-crown-6)][SbCl_6_]. The voids were calculated using the contact surface maps method, the probing radius and the step was set up to 0.2 Å.

Interesting features can be observed from the analysis of the voids in the [Rb(18-crown-6)][SbCl_6_] crystal at different pressures. Typically, at least 20% of the total unit-cell volume is unoccupied at ambient pressure even in non-porous molecular crystals (Wilson *et al.*, 2022 ▸). Definitely, in such cases, there is not enough free volume with a large radius to accommodate small solvent molecules, such as water. This is because the space is distributed over small interstitial sites around and between the molecules, making it inaccessible to guest molecules. Nevertheless, under ambient conditions, the crystal of [Rb(18-crown-6)][SbCl_6_] contains a significant number of voids between the molecules in the crystal confirmed by the presence of minimal H⋯Cl and H⋯H intermolecular contacts, which exceed the sum of their vdW radii. To estimate the volume occupied by molecules and voids in the crystal the data at various pressures were analyzed in *Mercury* (version 2023.3.0; Macrae *et al.*, 2020 ▸) using the contact surface maps method (the probing radius and step was set to 0.2 Å). Complete data which contains the total void volume unoccupied by molecules in the [Rb(18-crown-6)][SbCl_6_] crystal are presented in Table 4 ▸. Under ambient conditions (0.0 GPa), the void volume in a crystal is 468.5 Å^3^ or 26.5% of unit-cell volume, and it reduces under the pressure of 1.89 (3) GPa to 226.3 Å^3^ or 14.8%. Full data with the dependence of total void volume on pressure (*P*–void) are shown in Fig. 7 ▸. The graph clearly shows that it also consists of two linear sections with an inflection point of about ∼0.9 GPa which corresponds to a void volume of 317 Å^3^ or 19.5% of the unit-cell volume. Based on the position of the inflection point on the *P*–void graph, it appears that this point coincides with the average pressure value at the inflection points observed on the *P*–*a* and *P*–*c* dependence graphs. In addition, this inflection point also aligns with the average pressure value, which corresponds to the shortening of H⋯Cl contacts along different crystal axes to 2.95 Å.

This decrease in void volume with increasing pressure suggests that the rubidium atoms are rapidly approaching the center of the crown ether cavity. It is evident that as the pressure increases to 0.9 GPa, compressing the voids in the crystal becomes increasingly difficult, and the rubidium atom no longer approaches the center of the cavity as rapidly. Nevertheless, at a pressure of about 2 GPa, its position is so close to the center of the crown ether and the center of symmetry that it is almost indistinguishable by the X-ray diffraction method due to the significant overlap of electronic shells for disordered positions. Thus, we can conclude that the main factor determining the efficiency of compression of a given crystal under pressure is the presence of shortened intermolecular contacts in the crystal.

## 4. Conclusion

At relatively low pressures, there are no shortened intermolecular contacts in the crystal. The low value of pressure bulk modulus [9.1 (5) GPa] makes this hybrid organic–inorganic crystal comparable to various organic and organometallic compounds, for which dispersive and/or electrostatic interaction forces predominate. The high compression rate of [Rb(18-crown-6)][SbCl_6_] crystals at relatively low pressure can be attributed to the absence of shortened H⋯Cl and H⋯H intermolecular contacts in the crystal. The nonlinear dependence of unit-cell parameters on pressure can be explained by the influence of shortened intermolecular contacts in the crystal at pressures over 0.73 GPa. At pressures exceeding 0.9–1 GPa, steric repulsion forces become increasingly influential due to the shortening of several interatomic H⋯Cl and H⋯H contacts in the crystal to distances below the sum of their vdW radii. When the pressure is released on both dependences of the unit-cell parameter (*P*–*a* and *P*–*c*) below 0.9 GPa, hysteresis was observed and its influence on the disordered model of Rb atoms was demonstrated. Compression of the crystal, accompanied by a significant void reduction, leads to the rapid approach of Rb atoms toward the center of the crown ether cavity. The void reduction under pressure also demonstrates two linear sections with the inflection point at 0.9 GPa. Collectively, these factors play an important role in the compression and decompression behavior of the [Rb(18-crown-6)][SbCl_6_] crystal. To force the Rb atom inside the center of the crown ether cavity in this compound, pressures of about 2.5 GPa are required. Further studies of the high-pressure behavior of a similar compound with a larger Cs^+^ cation compressed in a DAC would be of considerable interest.

## Supplementary Material

Crystal structure: contains datablock(s) rbcrsbcl6_0k_100k, Rb0k0, Rb1k5, Rb2k9, Rb3k8, Rb6k0, Rb6k8, Rb8k3, Rb9k2, Rb9k8, Rb11k3, Rb12k5, Rb14k6, Rb16k1, Rb18k9, Rb12k3r, Rb9k5r, Rb7k6r, Rb3k0r, rb0k0r. DOI: 10.1107/S2052520624001586/px5059sup1.cif

Structure factors: contains datablock(s) rbcrsbcl6_0k_100k. DOI: 10.1107/S2052520624001586/px5059rbcrsbcl6_0k_100ksup2.hkl

Structure factors: contains datablock(s) Rb0k0. DOI: 10.1107/S2052520624001586/px5059Rb0k0sup3.hkl

Structure factors: contains datablock(s) Rb1k5. DOI: 10.1107/S2052520624001586/px5059Rb1k5sup4.hkl

Structure factors: contains datablock(s) Rb2k9. DOI: 10.1107/S2052520624001586/px5059Rb2k9sup5.hkl

Structure factors: contains datablock(s) Rb3k8. DOI: 10.1107/S2052520624001586/px5059Rb3k8sup6.hkl

Structure factors: contains datablock(s) Rb6k0. DOI: 10.1107/S2052520624001586/px5059Rb6k0sup7.hkl

Structure factors: contains datablock(s) Rb6k8. DOI: 10.1107/S2052520624001586/px5059Rb6k8sup8.hkl

Structure factors: contains datablock(s) Rb8k3. DOI: 10.1107/S2052520624001586/px5059Rb8k3sup9.hkl

Structure factors: contains datablock(s) Rb9k2. DOI: 10.1107/S2052520624001586/px5059Rb9k2sup10.hkl

Structure factors: contains datablock(s) Rb9k8. DOI: 10.1107/S2052520624001586/px5059Rb9k8sup11.hkl

Structure factors: contains datablock(s) Rb11k3. DOI: 10.1107/S2052520624001586/px5059Rb11k3sup12.hkl

Structure factors: contains datablock(s) Rb12k5. DOI: 10.1107/S2052520624001586/px5059Rb12k5sup13.hkl

Structure factors: contains datablock(s) Rb14k6. DOI: 10.1107/S2052520624001586/px5059Rb14k6sup14.hkl

Structure factors: contains datablock(s) Rb16k1. DOI: 10.1107/S2052520624001586/px5059Rb16k1sup15.hkl

Structure factors: contains datablock(s) Rb18k9. DOI: 10.1107/S2052520624001586/px5059Rb18k9sup16.hkl

Structure factors: contains datablock(s) Rb12k3r. DOI: 10.1107/S2052520624001586/px5059Rb12k3rsup17.hkl

Structure factors: contains datablock(s) Rb9k5r. DOI: 10.1107/S2052520624001586/px5059Rb9k5rsup18.hkl

Structure factors: contains datablock(s) Rb7k6r. DOI: 10.1107/S2052520624001586/px5059Rb7k6rsup19.hkl

Structure factors: contains datablock(s) Rb3k0r. DOI: 10.1107/S2052520624001586/px5059Rb3k0rsup20.hkl

Structure factors: contains datablock(s) rb0k0r. DOI: 10.1107/S2052520624001586/px5059rb0k0rsup21.hkl

CCDC references: 2333636, 2333637, 2333638, 2333639, 2333640, 2333641, 2333642, 2333643, 2333644, 2333645, 2333646, 2333647, 2333648, 2333649, 2333650, 2333651, 2333652, 2333653, 2333654, 2333655

## Figures and Tables

**Table 1:** Unit-cell parameters and refinement parameters in the [Rb(18-crown-6)][SbCl_6_] crystal at various pressures Suffix c indicates the rubidium atom was refined at the center of inversion. Suffix r indicates the data for crystal during the pressure release (decompression cycle).

Presure (GPa)	*a* (Å)	*c* (Å)	*V* (Å^3^)	*R*_int_	*R*2/*wR*2 [*I* > 2σ(*I*)]	GOF	Δρ (e Å^−3^)
0.0 (100 K)	14.0325 (7)	9.9333 (6)	1693.9 (2)	0.0282	0.0205/0.0451	1.054	0.54/−0.61
0.0 (293 K)	14.1212 (9)	10.2274 (9)	1766.2 (3)	0.0328	0.0274/0.0623	1.049	0.35/−0.64
0.15	14.0925 (13)	10.1233 (10)	1741.1 (4)	0.0431	0.0337/0.0592	1.044	0.45/−0.63
0.29	14.0661 (7)	10.0405 (5)	1720.4 (2)	0.0329	0.0275/0.0639	1.028	0.25/−0.43
0.38	14.0266 (7)	9.9794 (4)	1700.36 (18)	0.0327	0.0235/0.0511	1.059	0.23/−0.48
0.60	13.9661 (7)	9.8695 (5)	1667.15 (19)	0.0327	0.0222/0.0510	1.035	0.26/−0.47
0.68	13.9423 (7)	9.8301 (6)	1654.84 (19)	0.0283	0.0248/0.0571	1.054	0.28/−0.45
0.83	13.9035 (7)	9.7746 (5)	1636.35 (19)	0.0290	0.0237/0.0547	1.019	0.34/−0.46
0.92	13.8794 (7)	9.7474 (6)	1626.15 (19)	0.0261	0.0243/0.0555	1.084	0.29/−0.52
0.98	13.8732 (6)	9.7282 (5)	1621.49 (16)	0.0267	0.0291/0.0558	1.094	0.25/−0.46
1.13	13.8347 (6)	9.6719 (5)	1603.19 (16)	0.0265	0.0250/0.0579	1.087	0.42/−0.43
1.25	13.8133 (8)	9.6440 (5)	1593.6 (2)	0.0319	0.0254/0.0606	1.090	0.38/−0.49
1.46	13.7760 (8)	9.5808 (7)	1574.6 (2)	0.0294	0.0273/0.0601	1.070	0.34/−0.38
1.61	13.7392 (7)	9.5341 (6)	1558.58 (19)	0.0314	0.0274/0.0628	1.105	0.41/−0.53
1.89	13.6805 (8)	9.4433 (6)	1530.6 (2)	0.0344	0.0296/0.0677	1.032	0.40/−0.49
1.89*c*					0.0300/0.0680	1.036	0.40/−0.65
1.23r	13.8103 (10)	9.6615 (10)	1595.8 (3)	0.0465	0.0328/0.0715	1.003	0.46/−0.37
0.95r	13.8733 (11)	9.7541 (12)	1625.8 (3)	0.0463	0.0522/0.1105	1.043	0.67/−0.44
0.76r	13.9183 (9)	9.8453 (10)	1651.7 (3)	0.0440	0.0365/0.0835	1.065	0.58/−0.30
0.30r	14.0337 (12)	10.0671 (13)	1717.0 (4)	0.0405	0.0362/0.0752	1.017	0.18/−0.15
0.0r	14.1078 (8)	10.2256 (9)	1762.5 (3)	0.0491	0.0349/0.0861	1.071	0.81/−0.41

**Table 2:** Selected bond distances, contacts and torsion angles in the [Rb(18-crown-6)][SbCl_6_] crystal at various pressures Suffix c indicates the rubidium atom was refined at the center of inversion. Suffix r indicates the data obtained during the pressure release (decompression cycle).

Pressure (GPa)	*d*(Rb—O)(min, max) (Å)	*d*(Rb—Cl) (Å)	*d*(Rb–Rb)† (Å)	*d*(Sb—Cl) (Å)	H1*a*⋯Cl1, H1*b* ^i^⋯Cl1 (Å)	H2*b*⋯H2*a* ^i^ (Å)	O—C—C—O (°)
0 (at 100 K)	2.84676 (13), 2.9222 (13)	3.6834 (8)	0.9078 (14)	2.3743 (5)	2.97, 3.04	2.49	−68.9 (2)
0.0	2.8486 (17), 2.9354 (18)	3.7255 (13)	1.121 (2)	2.3741 (7)	3.08, 3.11	2.62	−68.9 (3)
0.15	2.846 (3), 2.927 (3)	3.7304 (17)	1.001 (3)	2.3641 (10)	3.03, 3.09	2.61	−70.3 (5)
0.29	2.845 (2), 2.921 (2)	3.7244 (13)	0.928 (2)	2.3693 (8)	3.02, 3.07	2.53	−69.5 (4)
0.38	2.8382 (19), 2.9105 (19)	3.7167 (11)	0.8840 (19)	2.3684 (7)	3.00, 3.05	2.50	−68.7 (3)
0.60	2.8387 (17), 2.9044 (18)	3.7077 (11)	0.7928 (19)	2.3701 (7)	2.97, 3.01	2.49	−68.9 (3)
0.68	2.835 (2), 2.899 (2)	3.7026 (13)	0.760 (2)	2.3726 (8)	2.95, 2.99	2.47	−69.1 (4)
0.83	2.836 (2), 2.896 (2)	3.6955 (13)	0.717 (2)	2.3717 (8)	2.93, 2.98	2.46	−69.1 (4)
0.92	2.831 (2), 2.8890 (2)	3.6932 (14)	0.696 (3)	2.3692 (7)	2.93, 2.96	2.45	−68.8 (4)
0.98	2.831 (2), 2.889 (2)	3.6891 (15)	0.682 (3)	2.3727 (8)	2.92, 2.96	2.43	−68.7 (4)
1.13	2.832 (2), 2.885 (2)	3.6841 (18)	0.634 (4)	2.3724 (8)	2.90, 2.95	2.41	−68.6 (4)
1.25	2.828 (2), 2.880 (3)	3.684 (2)	0.608 (5)	2.3729 (9)	2.89, 2.93	2.39	−68.4 (4)
1.46	2.832 (2), 2.879 (3)	3.687 (4)	0.534 (10)	2.3750 (9)	2.86, 2.92	2.38	−69.3 (4)
1.61	2.827 (2), 2.869 (3)	3.688 (6)	0.481 (13)	2.3734 (9)	2.85, 2.89	2.37	−69.1 (4)
1.89	2.822 (3), 2.857 (4)	3.681 (13)	0.40 (3)	2.3714 (10)	2.82, 2.86	2.33	−68.9 (5)
1.89c	2.833 (3)	3.8541 (11)	0	2.3713 (10)			−68.1 (4)
1.23r	2.827 (3), 2.881 (3)	3.678 (3)	0.634 (7)	2.3740 (11)	2.88, 2.93	2.43	−69.7 (5)
0.95r	2.835 (4), 2.895 (4)	3.688 (4)	0.705 (9)	2.3761 (16)	2.92, 2.95	2.43	−69.0 (8)
0.76r	2.834 (3), 2.901 (3)	3.687 (2)	0.804 (4)	2.3745 (12)	2.96, 2.97	2.47	−68.5 (5)
0.30r	2.839 (3), 2.917 (3)	3.703 (2)	1.000 (4)	2.3769 (13)	3.04, 3.05	2.557	−67.8 (6)
0.0r	2.846 (2), 2.933 (2)	3.7222 (16)	1.124 (3)	2.3761 (9)	3.08, 3.10	2.61	−68.5 (5)

Symmetry code (i): 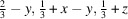 .

† The separation between two disordered positions of the Rb atom. (The deviation of the Rb from the crown ether plane is half of these values.)

**Table 3:** Short contacts in the [Rb(18-crown-6)][SbCl_6_] crystal at 1.89 (3) GPa

Atom 1	Atom 2	Distance (Å)	Symmetry code
Cl1	H1*a*	2.82	
Cl1	H1*b*	2.86	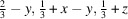
Cl1	H1*a*	2.96	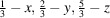
Cl1	H2*a*	2.90	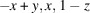
H1*b*	H2*a*	2.38	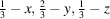
H2*b*	H2*a*	2.33	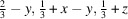

**Table 4:** Total void volume in the [Rb(18-crown-6)][SbCl_6_] crystal at various pressures Suffix r indicates the data obtained during the pressure release (decompression cycle).

Pressure (GPa)	Total void volume (Å^3^)	% of unit-cell volume
0.0	468.5	26.5
0.15	446.6	25.6
0.29	424.1	24.7
0.38	404.2	23.8
0.60	362.3	21.7
0.68	349.4	21.1
0.83	325.0	19.9
0.92	315.7	19.4
0.98	309.0	19.1
1.13	292.0	18.2
1.25	280.9	17.6
1.46	265.6	16.9
1.61	252.4	16.2
1.89	226.3	14.8
1.23r	280.7	17.6
0.95r	310.6	19.1
0.76r	346.2	21.0
0.30r	417.5	24.3
0.0r	466.3	26.5
